# High-resolution dataset of car, bike, and e-scooter sharing in Munich, Germany (2023–2025): Vehicle idling positions and derived trips

**DOI:** 10.1016/j.dib.2025.112156

**Published:** 2025-10-13

**Authors:** Tobias Herbst, Svetlana Zubareva, Markus Lienkamp

**Affiliations:** Technical University of Munich, TUM School of Engineering and Design, Department of Mobility Systems Engineering, Chair of Automotive Technology, Boltzmannstr. 15, 85748 Garching, Germany

**Keywords:** Shared mobility, Data scraping, GNSS tracking, Urban transportation

## Abstract

This dataset provides high-resolution, processed spatio-temporal data on shared mobility vehicles in Munich, Germany, collected between June 2023 and May 2025. It includes vehicle idling locations and periods, as well as derived trips for five providers across three mobility modes: two car-sharing, one bike-sharing, and two e-scooter-sharing systems. The dataset was created by processing vehicle availability data scraped at regular intervals from a public mobility platform. Idling locations were identified by clustering consecutive vehicle positions within a small spatial radius, while trips were inferred as movements between these idling periods. In addition, vehicle-level metadata such as model, color, fuel type, and timestamps of first and last appearance are included. The resulting dataset enables detailed investigations into, among others, fleet dynamics, user behavior, and temporal trends. All data are publicly accessible and partially validated against official trip records, offering a reliable resource for urban mobility research and multimodal transport analysis.

Specifications Table

**Table:** 

Subject	Engineering & Materials science
Specific subject area	GNSS-based Shared Mobility Dataset, Urban Transport, Multimodal Vehicle Tracking
Type of data	Mobility Data Table
Data collection	Vehicle availability data were scraped every three minutes from the public platform move.mvg.de between 1 June 2023, and 31 May 2025, using a custom Python script. The raw data include time-stamped positions of available shared vehicles from five providers. After storage in a PostgreSQL database, idling periods were identified based on spatial clustering, and trips were inferred as movements between idling periods with a minimum distance of 100 m and a maximum duration of 6 h.
Data source location	Data Collection Area: Location: Munich Metropolitan Area Longitude between 11.15° E and 11.9° E Latitude between 47.9° N and 48.4° N Data Storage Location: Institution: Technical University of Munich, TUM School of Engineering and Design, Chair of Automotive Technology City/Town/Region: 85748 Garching Country: Germany
Data accessibility	Repository name: Dataset of Idling Positions and Derived Trips of Shared Mobility Vehicles in Munich, Germany (2023–2025) Data identification number: 10.5281/zenodo.16947276 Direct URL to data: https://zenodo.org/records/16947276
Related research article	none

## 1 Value of the Data

• **High-resolution idling and trip data:** The dataset contains processed spatio-temporal data on vehicle idling locations and periods, as well as derived trips, based on three-minute interval observations with meter-level spatial precision. This granularity enables detailed analysis of vehicle availability, idle durations, and movement patterns across urban space.
• **Extensive two-year observation period:** Data were collected continuously from June 2023 to May 2025, allowing for robust temporal analyses. Researchers can explore seasonal effects, long-term behavioral trends, and year-over-year changes in shared mobility usage and fleet composition.
• **Across providers and modes:** The dataset covers five major providers spanning three mobility modes: two car-sharing, one bike-sharing, and two e-scooter-sharing. This broad multimodal scope captures a significant share of Munich’s shared mobility landscape and enables direct comparisons between providers within each mode.
• **Vehicle-specific attributes**: Each vehicle is individually described by its model, color, fuel type, and the timestamps of its first and last appearance. These attributes enable analyses of fleet composition, vehicle lifespans, and the impact of vehicle characteristics on usage intensity.
• **Partially validated against official records:** Trip-level data have been matched with official records, confirming the reliability of the derived trips.
• **Ready for reuse and extension:** The dataset is publicly available in a structured format, making it suitable for reuse in transport modeling, geospatial analysis, machine learning, and urban planning. It can be enriched with contextual data such as weather, events, or infrastructure, enabling studies on, for example, the influence of weather on shared mobility usage and the identification of suitable locations for shared mobility parking zones.

## 2 Background

Despite growing interest in shared mobility, publicly available datasets remain sparse, with limited modal representation, geographic diversity, and spatial granularity. Most open datasets originate from U.S. cities and focus on docked bike-sharing systems, such as those in Chicago [1], New York [2], Washington, D.C [3], Philadelphia [4], Boston [5], and the Bay Area [6]. These provide station-to-station trip data with timestamps, vehicle IDs, and basic customer information. A few cities, such as Austin [7] (bikes, e-scooters) and Chicago [8] (e-scooters), publish data on free-floating systems, but these are spatially aggregated and lack vehicle-level detail.

Munich [9] provides raw trip data from a free-floating bike-sharing system, but without vehicle identifiers. A web-scraped dataset on car-sharing services is available for Turin [10], while for Berlin [11] and other European cities [12], web-scraped datasets on bike-sharing are available; all of these datasets cover less than one year of recorded data.

Due to these limitations, existing datasets do not support analysis of fine-grained idle times and locations, the influence of vehicle-specific attributes, or the interaction of multiple operators and modes. The dataset presented here addresses these gaps, offering two years of high-resolution, vehicle-level data across five providers and three shared mobility modes.

## 3 Data Description

This dataset provides high-resolution information on shared mobility vehicles in Munich, Germany, collected between 1 June 2023 and 31 May 2025. It covers five providers across three modes: Miles and ShareNow (car-sharing), MVG Rad (bike-sharing), and TIER and VOI (e-scooter-sharing). The data include processed data on vehicle idle locations and periods (Section 2.1), as well as derived trip information (Section 2.2), recorded with meter-level spatial precision and three-minute temporal resolution. Additional vehicle-specific information, such as model specifications, fuel type, and the timestamps of the first and last sightings, is also available (Section 2.3). Each provider’s service area geometry is provided separately (Section 2.4). All processed datasets are publicly accessible through an online repository [13].

### 3.1 Idling_{provider}.parquet.gz

The idling datasets show where and for how long each vehicle was available (Table 1). Separate files are provided for each of the five mobility providers, following the file naming convention idling_{provider}.parquet.gz.

**Table 1:** Data structure of idling_{provider}.parquet.gz.

Column	Data Type	Description
Id	string	vehicle ID
Lat	float	latitude (EPSG:4326) of the vehicle’s idling location
lon	float	longitude (EPSG:4326) of the vehicle’s idling location
starttime	int	unix timestamp of the vehicle’s idling start time
endtime	int	unix timestamp of the vehicle’s idling end time

The two e-scooter data sets have the most entries (Table 2). Both the respective fleet size (Table 5) and data availability (Fig. 7) must be taken into account.

**Table 2:** Data overview of idling_{provider}.parquet.gz.

Provider	Type	Number of Idling Periods
Miles	car-sharing	2873,693
MVG Rad	bike-sharing	1582,172
ShareNow	car-sharing	1348,692
TIER	e-scooter-sharing	3011,856
VOI	e-scooter-sharing	5454,555

**Fig. 1:**
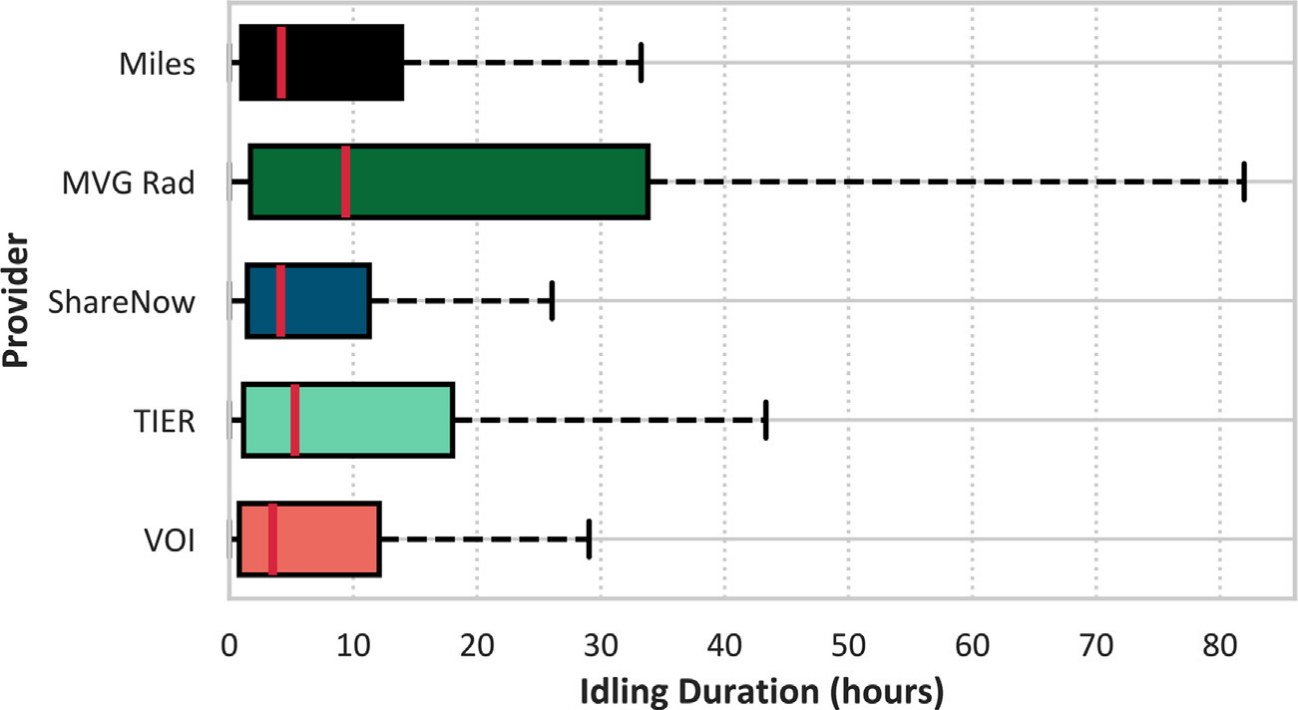
Boxplot of idling durations per provider.

MVG Rad exhibits the longest idling durations, with a median of nearly 9.5 h and a wide interquartile range. In contrast, vehicles from car-sharing and e-scooter providers show considerably shorter idling times, with median values ranging between 3.5 and 5.5 h (Fig. 1).

### 3.2 Trips_{provider}.parquet.gz

The trip datasets contain individual vehicle trips derived from the idling data. Only movements covering a minimum distance of 100 m, as determined based on the threshold in Fig. 6, and lasting no longer than 6 h are included. Each trip entry includes the vehicle ID, start time and location, and end time and location (Table 3). No information is available about the route taken or pricing. The trip files are provided separately for each of the five mobility providers, following the file naming convention trips_{provider}.parquet.gz.

**Table 3:** Data structure of trips_{provider}.parquet.gz.

Column	Data Type	Description
id	str	vehicle ID
startlat	float	latitude (EPSG:4326) of the trip's departure position
startlon	float	longitude (EPSG:4326) of the trip's departure position
starttime	int	unix timestamp of departure
endlat	float	latitude (EPSG:4326) of the trip's arrival position
endlon	float	longitude (EPSG:4326) of the trip's arrival position
endtime	int	unix timestamp of arrival

**Fig. 2:**
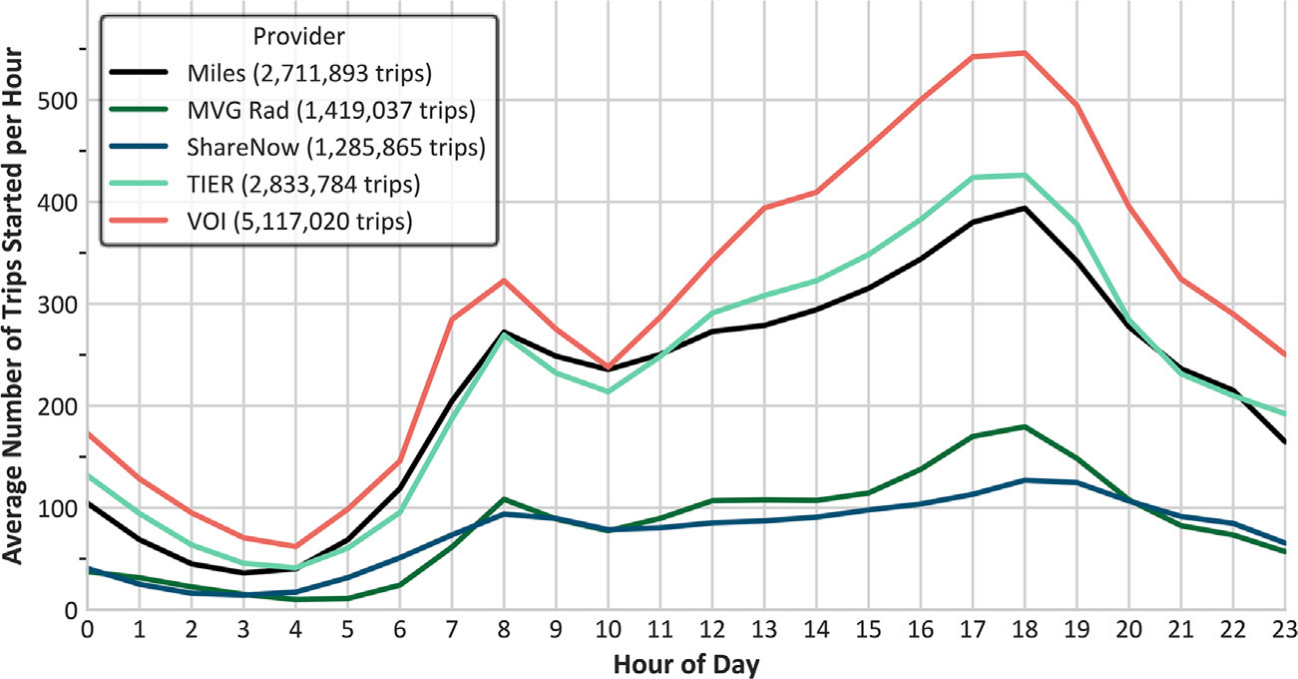
Average number of trips started each hour of the day, based on daily data. Each hourly bin includes trips that began at any time from the start of the hour up to, but not including, the next hour.

Across all providers, most trips occur in the afternoon and evening, with a smaller peak in the morning (Fig. 2). The datasets containing e-scooters account for the highest number of trips.

**Fig. 3:**
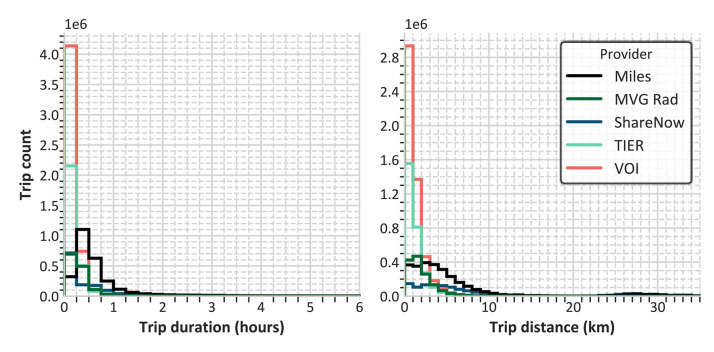
Histogram of trip durations and distances across all providers.

Trips with e-scooters and bicycles tend to be shorter both in terms of duration and distance compared to car-sharing trips (Fig. 3).

### 3.3 Vehicles_{provider}.parquet.gz

These datasets contain supplementary information for each vehicle, including its model, color, and fuel type (Table 4). The entries for color and fuel type are in German, as they reflect the original language of the source data. It also records the timestamps of when the vehicle first and last appeared in the raw data. Using the vehicle ID, this dataset can be linked to the Idling (Section 2.1) and Trips Dataset (Section 2.2). Separate files are provided for each of the five mobility providers, following the file naming convention vehicles_{provider}.parquet.gz.

**Table 4:** Data structure of vehicles_{provider}.parquet.gz.

Column	Data Type	Description
id	str	vehicle ID
vehicle_type	str	vehicle’s model specification
fuel_type	str	vehicle's primary energy source
color	str	vehicle color
time_first_seen	int	unix timestamp of vehicle’s first appearance in the data
time_last_seen	int	unix timestamp of vehicle’s last appearance in the data

During the observation period, the two e-scooter providers recorded the highest number of unique vehicles (Table 5). However, the median vehicle age differs significantly between them. It is important to note that the reported median service age refers only to the two-year observation window, not the vehicle's total lifespan. The two car-sharing providers differ in fleet size but show similarities in electrification rate and vehicle age. The bike-sharing fleet remained largely unchanged over the two years.

**Table 5:** Fleet size, electrification rate, and median service age (in days) by provider over the two-year observation period.

Provider	Number of Vehicles	Electrification Rate	Median Service Age (Days)
Miles	5012	5.7 %	330
MVG Rad	3796	0 %	725
ShareNow	1727	4.7 %	358
TIER	6702	100 %	521
VOI	9241	100 %	92

### 3.4 Service_area_{provider}.parquet.gz

This dataset defines the boundaries of the service area (Table 6). The areas included in the dataset represent the state of the service area at the end of the recording phase. Within these boundaries, there are additional no-parking zones for e-scooters, such as those located in parks or city centers. However, no specific data is available for these no-parking zones or for any changes that may have occurred during the data collection period. Users should note that analyses of e-scooter distribution may be affected by the absence of this information.

**Table 6:** Data structure of service_area_{provider}.parquet.gz.

Column	Data Type	Description
provider	str	name of the provider
geom_service_area	str	multipolygon of provider’s service area (EPSG:4326)

## 4 Experimental Design, Materials and Methods

This chapter details the data collection and processing methods used to generate the idling and trips datasets. It covers how raw vehicle positions were gathered (Section 3.1), how idling periods were identified (Section 3.2), and how trips were inferred from these periods (Section 3.3).

### 4.1 Raw data collection

The raw data were sourced from the website move.mvg.de, which displayed the real-time positions of available vehicles and is no longer accessible. A Python script scraped the provider's website every three minutes to collect vehicle position data at meter-level precision, accessing a JSON endpoint and parsing the response automatically Table 7.

**Table 7:** Data structure of the raw data.

Column	Data Type	Description
id	string	vehicle ID
lat	float	latitude (EPSG:4326) of the vehicle’s position
lon	float	longitude (EPSG:4326) of the vehicle’s position
time	int	unix timestamp of request

**Fig. 4:**
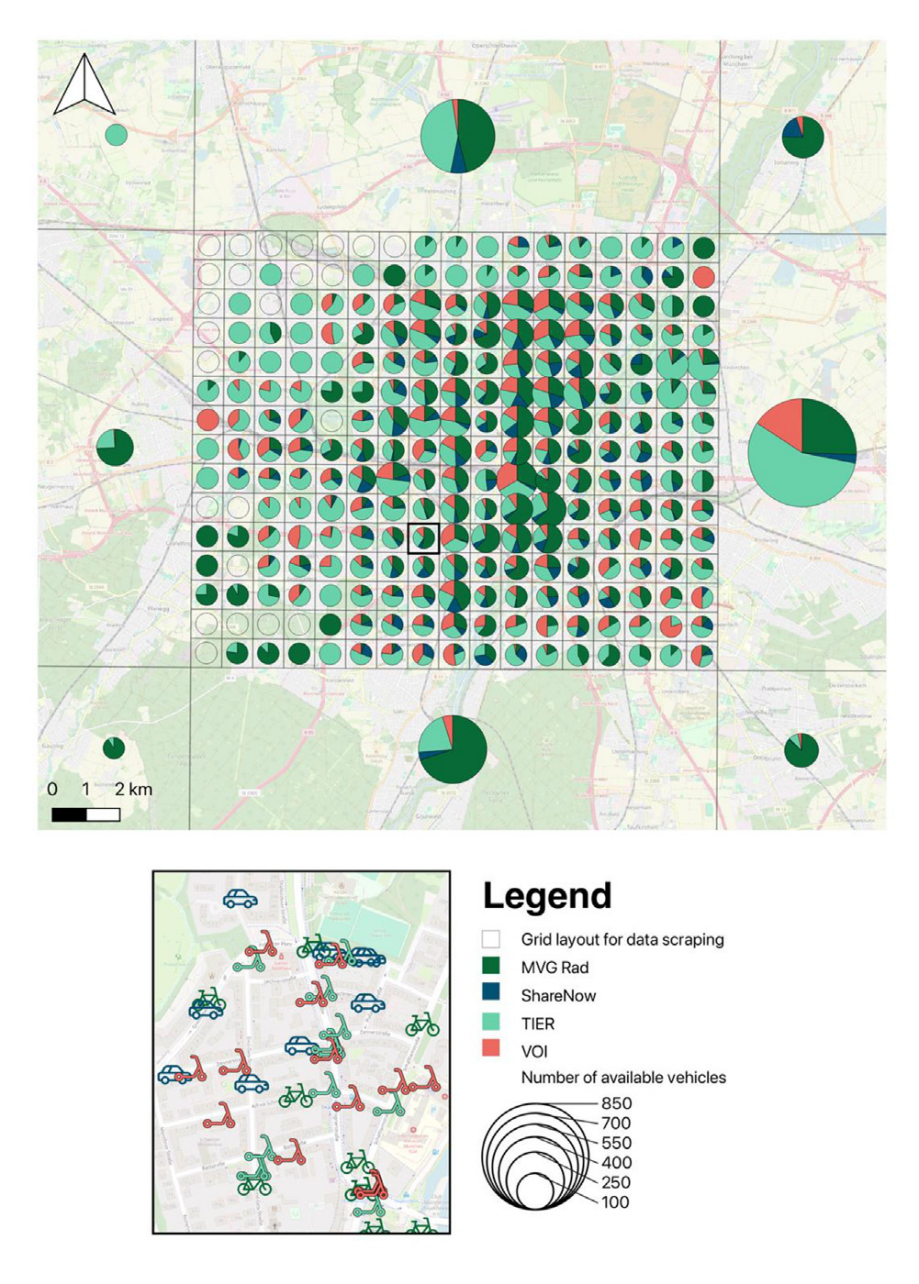
The figure shows the grid cells queried by the script, including the number of vehicles and the provider’s share in each cell as of 2023–06–01 00:00. In the urban area, each cell measures 1200 *m* × 1200 m; in the surrounding area, four rectangles cover the remaining region from 47.9° N to 48.4° N and from 11.15° E to 11.9° E (for a clearer representation, the 100 m overlap per cell is not shown). To visualize the distribution among providers, the vehicles are aggregated per cell. The original dataset contains the raw position data at meter-level resolution. Inset: original position data without aggregation at the cell level.

Due to website limitations preventing simultaneous retrieval of all vehicles, the Munich metropolitan area was divided into grid cells, and within each cell, all vehicle locations were scraped without aggregation (Fig. 4). Each cell in the city center measured 1200 m by 1200 m with a 100 m overlap to adjacent cells. In contrast, the city’s outskirts and surrounding areas were covered using larger polygons, as these regions typically have lower vehicle densities. Duplicate entries arising from the overlaps were removed before storing the cleaned data in a PostgreSQL database. The queried URLs are available in the project repository [13].

**Fig. 5:**
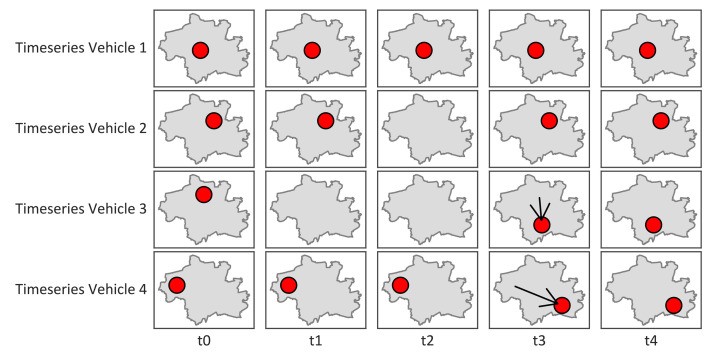
Exemplary spatio-temporal patterns in vehicle position data. Each row represents a single vehicle’s location over time when available: Vehicle 1 – Continuous availability without position change; Vehicle 2 – Temporary disappearance, then reappears at the same position; Vehicle 3 – Temporary disappearance, then reappears at a different position; Vehicle 4 – Continuous availability with changing position.

This structured data collection approach enables the tracking of vehicle behavior over time, laying the groundwork for analyzing mobility patterns and idling behavior. A vehicle may remain at the same location across multiple time steps if it is idling. Over time, vehicles typically show mobility through changes in position or temporary disappearance during trips or reservations. Fig. 5 illustrates the four most prevalent patterns of vehicle idling and movement over time.

**Fig. 6:**
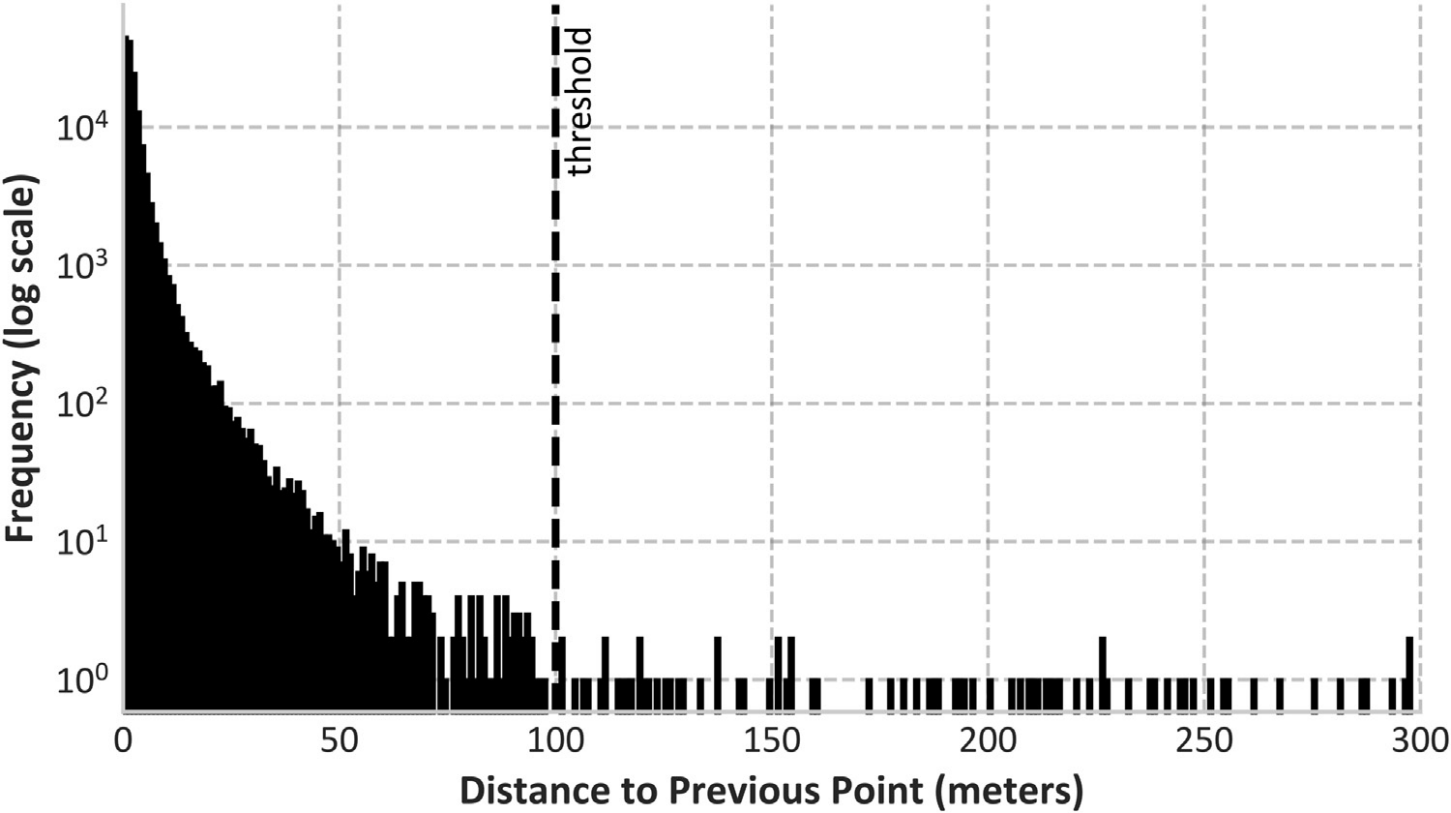
Histogram with 1 m bins of distances between successive entries (up to 300 m) of one exemplary e-scooter (id = ‘voiescootere7eu’). The threshold shown indicates the minimum distance that must lie between two consecutive points to be determined as a trip.

The position changes contained in the raw data are not always as pronounced as shown in Fig. 5. This is because vehicles regularly transmit status updates that include new position measurements. These measurements are influenced by GPS jitter, resulting in numerous small position shifts of no more than several meters (Fig. 6).

### 4.2 Creation of the idling dataset

The locations and durations of idling periods were derived for each vehicle individually, based on the characteristics of the raw data. The pattern shown for Vehicle 2 in Fig. 5 is frequently observed. However, this pattern may result from one of three potential causes, and the true reason remains unclear: (1) the vehicle is reserved and therefore unavailable; (2) the vehicle is rented and returned to the same location; or (3) the vehicle is missing for one or more timesteps in the scraped dataset due to an error in the data pipeline.

Due to these uncertainties, idling periods are identified solely using a distance-based criterion. As a result, reservations and round-trip journeys are not represented in the dataset.

A new idling period is initiated whenever the distance between two consecutive vehicle positions exceeds 100 m. This threshold was determined empirically after inspecting the data shown in Fig. 6, which indicates that most short-range positional changes were caused by GPS jitter rather than actual movement. For each idling period, the centroid of all associated points is computed to approximate the vehicle’s location during that period.

### 4.3 Trip detection

Trips are derived directly from the idling dataset: each trip represents the transition from one idling location to another. The start and end positions of a trip correspond to the respective locations of the idling periods, while the trip’s start and end times are defined by the end and beginning of the preceding and following idling periods, respectively.

To ensure meaningful and representative movement, each trip must meet two criteria: (1) a minimum distance of 100 m between the centroids of the two idling locations, and (2) a maximum duration of 6 h.

## Limitations

**Fig. 7:**
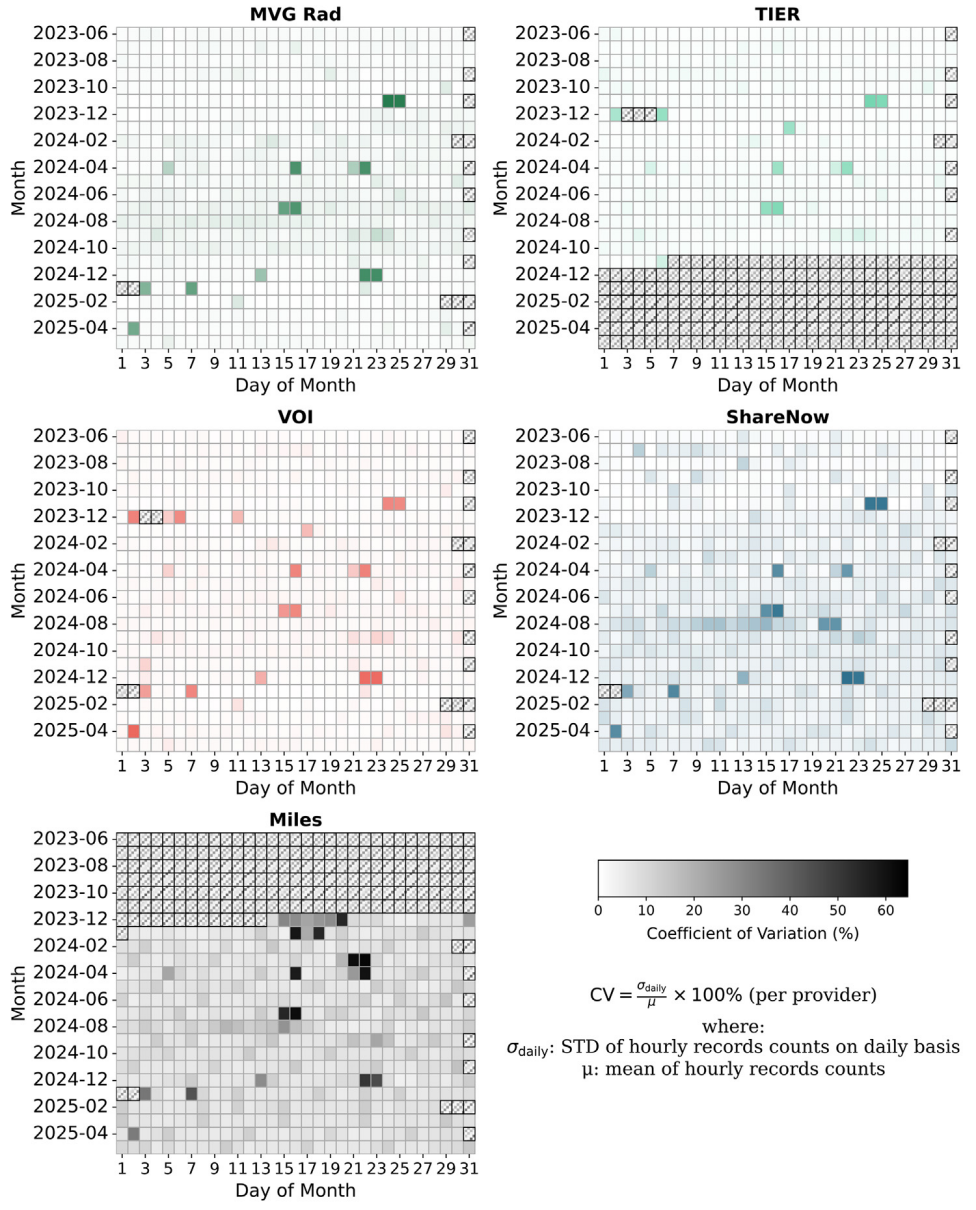
Coefficient of Variation (CV) of data entries on a daily basis per provider.

Periods affected by vehicle-independent scraping issues and therefore reduced accuracy are indicated in Fig. 7.

For Miles, data is only available from December 14, 2023, and for TIER, only until November 6, 2024 (Fig. 7). Some ShareNow vehicle IDs, especially those starting with “sharenowHH-SN” (473 IDs), “sharenowHH—N” (2 IDs), and “sharenowHH-SX” (268 IDs), frequently show trips lasting under five minutes. Trips with <100 m are missing in all datasets. Other providers than the five also operate in Munich.

Data quality is assessed by comparing with MVG Rad's official trip data [9]. Trips are matched if start and end points differ by at most 100 m and times by at most 20 min. Of 100,276 official trips in June 2023, 88,407 (88.2 %) are included in trips_mvgrad.parquet.gz. Of the remaining 11,869 trips, 7244 are shorter than 100 m, 925 longer than 6 h, and 1709 longer than 50 km. In the same period, trips_mvgrad.parquet.gz contains 7106 trips (7.4 %) with no corresponding official trip. The impact of rebalancing cannot be quantified due to a lack of official data.

## Ethics Statement

The authors confirm that they have read and adhere to the ethical requirements for publication in Data in Brief. Furthermore, the current work does not involve human subjects, animal experiments, or any data collected from social media platforms.

## Credit Author Statement

**Tobias Herbst:** Conceptualization, Methodology, Software, Validation, Investigation, Data Curation, Writing – Original Draft, Visualization. **Svetlana Zubareva:** Formal Analysis, Writing – Original Draft, Visualization. Markus Lienkamp: Resources, Writing – Review & Editing, Supervision, Funding acquisition

## Declaration of Generative AI and AI-Assisted Technologies in the Writing Process

During the preparation of this work the authors used chatGPT and Grammarly to improve language and readability. After using this service, the authors reviewed and edited the content as needed and take full responsibility for the content of the publication.

## Data Availability

ZenodoDataset of Idling Positions and Derived Trips of Shared Mobility Vehicles in Munich, Germany (2023–2025) (Original data)
